# Avoidance of the Plant Hormone Cis-Jasmone by *Aedes aegypti* Depends On Mosquito Age in Both Plant and Human Odor Backgrounds

**DOI:** 10.1007/s10886-021-01299-2

**Published:** 2021-08-31

**Authors:** Jetske G. de Boer, Aron P. S. Kuiper, Joeri Groot, Joop J. A. van Loon

**Affiliations:** 1 Department of Terrestrial Ecology, Netherlands Institute of Ecology (NIOO-KNAW), Wageningen, The Netherlands; 2 Laboratory of Entomology, Wageningen University, Wageningen, The Netherlands

**Keywords:** Plant defense, Plant volatiles, Jasmonic acid, Human odor, Repellent, Aversion, DEET

## Abstract

Adults of many mosquito species feed on plants to obtain metabolic energy and to enhance reproduction. Mosquitoes primarily rely on olfaction to locate plants and are known to respond to a range of plant volatiles. We studied the olfactory response of the yellow fever mosquito *Aedes aegypti* to methyl jasmonate (MeJA) and *cis*-jasmone (CiJA), volatile compounds originating from the octadecanoid signaling pathway that plays a key role in plant defense against herbivores. Specifically, we investigated how *Ae. aegypti* of different ages responded to elevated levels of CiJA in two attractive odor contexts, either derived from Lima bean plants or human skin. *Aedes aegypti* females landed significantly less often on a surface with CiJA and MeJA compared to the solvent control, CiJA exerting a stronger reduction in landing than MeJA. Odor context (plant or human) had no significant main effect on the olfactory responses of *Ae. aegypti* females to CiJA. Mosquito age significantly affected the olfactory response, older females (7–9 d) responding more strongly to elevated levels of CiJA than young females (1–3 d) in either odor context. Our results show that avoidance of CiJA by *Ae. aegypti* is independent of odor background, suggesting that jasmonates are inherently aversive cues to these mosquitoes. We propose that avoidance of plants with elevated levels of jasmonates is adaptive to mosquitoes to reduce the risk of encountering predators that is higher on these plants, i.e. by avoiding ‘enemy-dense-space’.

## Introduction

Adults of many mosquito species feed on plant fluids to obtain metabolic energy and to enhance reproduction, and plants provide the only source of energy for male mosquitoes (Barredo and DeGennaro 2020; Foster 1995; Peach and Gries 2020). Floral nectar is the most widely used source of plant carbohydrates, but mosquitoes may also feed on extrafloral nectar or tree sap. Besides carbohydrates, mosquitoes may derive other important compounds from plants, including amino acids (Peach and Gries 2020; Vrzal et al. 2010). Recent studies have shown that plant-feeding is more common than previously thought among important disease-vectoring mosquitoes, including *Anopheles*, *Aedes,* and *Culex* species (e.g. Nyasembe et al. 2018; Olson et al. 2020; Sissoko et al. 2019). Fitness benefits for these mosquitoes can be plant species-specific due to differences in nectar composition. For example, survival and reproduction of female *Anopheles gambiae* mosquitoes were higher on plants with higher total nectar sugar concentrations (Manda et al. 2007a). These differential fitness benefits were reflected in the clear preferences of *An. gambiae* mosquitoes for these plant species (Manda et al. 2007b).

Mosquitoes primarily rely on visual and olfactory cues to locate plants, and mosquito preferences for certain plant species can be mediated by the composition of plant volatile blends (Lahondère et al. 2020; Nikbakhtzadeh et al. 2014; Nyasembe et al. 2018; von Oppen et al. 2015). Although mosquito attraction to plants has received far less attention than attraction to vertebrate hosts, it presents a new opportunity to develop mosquito lures for population monitoring or control (Dormont et al. 2021; Foster 2008; Nyasembe and Torto 2014). In contrast, using plants and plant-derived compounds to repel mosquitoes has a much longer history. Plants continue to be exploited in the search for natural and safe mosquito repellents to replace synthetic compounds such as DEET (*N,N*-diethyl-3-methylbenzamide) that remains the most widely used insect repellent to date (Chellappandian et al. 2018; Grison et al. 2020). In this respect, Xu et al. (2014) made an interesting discovery when they demonstrated that the plant compound methyl jasmonate (MeJA) activates the same odorant receptor as DEET in the southern house mosquito *Culex quinquefasciatus*. MeJA and DEET share similar structural motifs and it was suggested that MeJA may be the natural ligand for this odorant receptor. At a behavioral level, *C. quinquefasciatus* is repelled by MeJA as well as by the closely related compound methyl dihydrojasmonate (Xu et al. 2014; Zeng et al. 2018), and the tick *Ixodes ricinus* is also repelled by MeJA (Garboui et al. 2007).

Here, we address the question of why blood-feeding arthropods avoid these plant-derived compounds. We propose an explanation based on the role of jasmonates in plant defense. Jasmonic acid (JA) is a plant hormone that plays a key role in defense against herbivores (Gfeller et al. 2010; Wasternack 2015). Activation of the JA-pathway in plants leads to increased direct as well as indirect defense against herbivores. Direct defense reduces herbivore performance on and attraction to these plants (e.g. Sobhy et al. 2020). Upregulation of JA also triggers the emission of herbivore-induced plant volatiles that attract natural enemies of herbivores and thereby serves as an indirect defense mechanism (Turlings and Erb 2018). Herbivorous insects and their natural enemies also respond behaviorally to MeJA and CiJA. For example, thrips and aphids avoid these compounds, while parasitoid wasps and predatory insects are attracted (Birkett et al. 2000; Egger et al. 2016; Ibrahim et al. 2005; Sun et al. 2020). To mosquitoes, elevated levels of jasmonates might signal a higher risk of encountering natural enemies, i.e. be associated with enemy-dense-space. In this scenario, we expect mosquitoes to avoid elevated levels of jasmonates, regardless of the context in which these volatiles are presented.

In this study, we set up a series of experiments with the yellow fever mosquito *Aedes aegypti* to test this explanation. We also included mosquito age because mosquito olfactory responses may depend on their age and younger mosquitoes are more likely to search for a sugar-meal than older ones (Foster and Takken 2004; Tallon et al. 2019). Our objectives were to (1) determine whether *Ae. aegypti* avoids elevated levels of MeJA and CiJA in a landing assay, and (2) investigate whether odor context (plant or human) and mosquito age affect the olfactory response of female *Ae. aegypti* to CiJA.

## Methods

### Mosquito Rearing

*Aedes aegypti* mosquitoes were reared in a climate-controlled room (28 ± 1 °C, 70 ± 5% RH, and L:D 12 h:12 h). Adults were kept in 30 × 30 × 30 cm insect cages (Megaview, Taichung, Taiwan) with 6% glucose solution. Two to three times per week, human blood (Sanquin, Blood Bank Nijmegen, The Netherlands) was offered in a membrane feeder (Hemotek®, Blackburn, U.K.), covered with Parafilm, with a worn nylon sock positioned around the membrane to stimulate natural feeding behavior. Eggs were laid on wet filter paper. Eggs were collected three times per week and transferred into plastic buckets (19 × 19 × 21 cm) with water and Liquifry (Interpet, U.K.), covered with nylon mesh. The larvae were fed Tetramin® (Tetra, Melle, Germany) fish food three times a week until they pupated. Newly emerged adults were transferred into insect cages and supplied with glucose until use. Female mosquitoes were collected the day before an experiment and kept with tap water only. Mosquitoes were used in experiments approximately 7–9 d after adult emergence (further referred to as ‘old’), except for the ‘young’ mosquitoes used in the Y-tube olfactometer, which were selected from the rearing buckets 1–3 d after adult emergence and did not receive any sugar before testing. We assumed that all females were mated.

### Landing Assay

Avoidance of *Ae. aegypti* females by *cis*-jasmone and methyl jasmonate was tested in a landing assay according to methods described by Menger et al. (2014). The flight arena consisted of a 30 × 30 × 30 cm insect cage (Megaview, Taichung, Taiwan). A heated surface (kept at 34 ± 2 °C), moisture, and host odor were presented underneath the bottom of the cage to attract mosquitoes to the landing area. As a mimic of human odor, nylon strips incubated with a blend of five synthetic compounds were used, this Mbita-5 blend is known to be attractive to *Ae. aegypti* females (Visser et al. 2020). To mimic breathing, CO_2_ (5%) was intermittently blown into the flight arena at 4 s intervals for 1 s (flow rate 80 ml/min). The test compounds *cis*-jasmone (CiJA, 94% purity, Alfa Aeser, Karlsruhe, Germany) and methyl jasmonate (MeJA, 95% purity, Sigma Aldrich, Steinheim, Germany) were dissolved in ethanol (100%, Merck, Darmstadt, Germany). We compared these compounds to a negative control of ethanol (EtOH). Each compound was applied onto nylon strips (10 × 10 cm) by incubating each strip in a 4 ml vial with 2 ml of the solution and storing vials at 4 °C until use. Before testing, excess fluid was drained from the treated strips onto filter paper, and one strip was placed at the bottom in the center of the flight arena, positioned around and above the attractive lure and the heated surface. Each compound was tested in separate insect cages to prevent cross contamination of test compounds and controls. Per test, 4–10 female mosquitoes (average 7.25 mosquitoes across all experiments) were released into the flight arena by placing a release container in the corner of the cage. After two min of acclimatization, landings were counted for eight min, as defined in Menger et al. (2014). We tested CiJA and MeJA at two concentrations in two separate experimental series (0.1% and 1%), each with EtOH as a negative control. Eight and seven replicates of each treatment were done respectively in the first and second series of experiments.

Experiments were conducted in the morning inside a climatized room (set at 27 ± 1 °C) with artificial light (around 100 lx inside the flight arena). The air temperature inside the cage and relative humidity in the room were recorded and were 27.4 ± 0.10 °C (mean ± SE) and 79.5 ± 1.4% RH in the first series of experiments, and 26.8 ± 0.19 °C and 78.2 ± 1.6% RH in the second.

### Y-tube Olfactometer Experiments

To evaluate the olfactory response of *Ae. aegypti* to CiJA in the context of human odor or plant odor, we used a Y-tube olfactometer. The design of this olfactometer was based on a Y-tube described by Geier and Boeckh (1999). The Y-shaped tube was made of glass and had an inner diameter of 6 cm (basal arm 60 cm long, oblique parts 10 cm long, parallel arms 27 cm long). Each arm was connected to a mosquito capture cage consisting of a plexiglass cylinder (10 cm long and 7 cm inner diameter), closed with metal mesh on the upwind-end and a metal mesh door on the downwind-end. Each capture cage was connected to a series of two odor chambers, consisting of plexiglass cylinders of the same size and with metal mesh on the upwind-end but left open on the downwind-end. The odor chambers at the upwind end were covered with lids, through which CO_2_ (100%) and activated charcoal-filtered air (28.5 ± 2 °C, 60 ± 7% RH) were led at flow rates of *ca.* 50 mL/min and 34 L/min respectively, resulting in an air velocity of approximately 0.2 m/s in each arm. Mosquitoes were released from a plexiglass cylinder positioned at the downwind basal arm of the Y-tube olfactometer. Release cages were closed with metal mesh on one side and a metal grid door on the side opening towards the olfactometer. The different compartments of the Y-tube olfactometer were connected airtight with 2.5 cm wide washers with rubber rings on the inside to cover connecting points. The glass tube was cleaned with acetone between the different experiments and plexiglass components were cleaned with non-fragrant detergent every day.

The odor context was created by offering plant or human odor in both arms of the olfactometer in the first set of odor chambers. Non-flowering Lima bean (*Phaseolus lunatus* L. cv. Jackson Wonder Bush) plants of 4–5 weeks old were used. Lima bean is an important model plant species in induced defense against herbivores (Koch et al. 1999), has extrafloral nectaries and it is known to emit CiJA under herbivore attack, also from excised plant parts (e.g. Kost and Heil 2006). Moreover, a recent field study showed that plants of the Fabaceae family provide important sources of sugar for *Ae. aegypti* in Kenya (Wanjiku et al. 2021). We used two primary leaves and one compound leaf (excised at the main stem of the plant) as an odor source, and freshly excised plant material was used each day. Human odor consisted of six pairs of nylon socks worn by volunteers overnight (97% nylon, 3% elastane, 20 denier, HEMA, The Netherlands). Twelve equivalent human odor sources were created by cutting each worn sock into six strips – obtaining twelve strips – and combining one strip per volunteer into a bundle. Each bundle of sock strips was wrapped in aluminum foil and kept at -20 °C until and between experiments. CiJA was presented in the second set of odor chambers by pipetting 100 µl of either a 0.1% or 1% solution in EtOH on a filter paper (Ø 45 mm AllPaper b.v., The Netherlands) that was hung inside an odor chamber on one side. A filter paper with 100 µl EtOH was used as a control on the other side. We used new filter papers with CiJA or EtOH for each set of mosquitoes. Per experimental day, we started with either plant or human odor, and the order of CiJA concentration tested was randomized. To minimize any effect of side bias, treatments were tested on both sides of the olfactometer equally often. Six replicates of this experiment were done on six different days. A higher concentration of 10% CiJA combined with plant or human odor was tested on two additional days for a total of six replicates. We verified the attraction of *Ae. aegypti* to plant and human odor in this set-up in a separate control experiment by presenting (1) a set of Lima bean leaves against nothing, (2) a bundle of worn sock strips against a bundle of clean sock strips, or (3) a set of Lima bean leaves against a bundle of worn sock strips. Female mosquitoes of different ages (1–3 d or 7–9 d after adult emergence, further referred to as young and old respectively) were tested on different days in all experiments.

For each test, a release cage with approximately 20 female mosquitoes was connected to the olfactometer. Mosquitoes were allowed to settle for 90 s inside the release cage before the door to the Y-tube olfactometer was removed. Mosquitoes then flew freely in the olfactometer for 3 min, after which the doors of the capture cages and release cage were closed. We counted the number of mosquitoes inside the release cage, Y-tube, and inside each capture cage. Finally, all mosquitoes were removed with a vacuum cleaner.

All experiments were carried out in the afternoon. The olfactometer was positioned inside a dark climatized room (28.5 ± 2 °C, 65 ± 5% RH), with two 60 W lights, positioned approximately 50 cm above the olfactometer. The olfactometer was surrounded by white paper to minimize effects of visual stimuli that might lead to a side bias.

### Statistical Analyses

The effect of jasmonates on mosquito behavior in the landing assay was assessed by counting the number of landings per group of mosquitoes. Separate generalized linear models (GLM) were used per experimental series because low and high concentrations were tested on different days. We evaluated the effect of treatment (CiJA, MeJA, and EtOH) on the number of landings. The number of mosquitoes per run, temperature inside the cage, and RH were used as covariates in the model when they were significant (P < 0.05). Landing data were overdispersed and we, therefore, used a negative binomial distribution (log-link function) (R package “pscl”). *P-*values for variables were obtained by comparing models with likelihood ratio tests. Pairwise comparisons between treatments were made by computing estimated marginal means from the final models using R package “emmeans”.

Results of the Y-tube olfactometer assays were first evaluated with binomial tests to assess whether mosquito choices between each set of treatment and control odor sources differed from a 50:50 distribution. This was done with the mosquitoes trapped on the treatment and the control odor source, summed over six replicates. We then assessed the impact of odor context (plant or human) and CiJA concentration on *Ae. aegypti* olfactory responses, using separate GLMs for young and old mosquitoes. Finally, the effect of mosquito age was evaluated along with CiJA concentration in a combined model, i.e. irrespective of odor context. *P-*values for each explanatory variable and their interaction were obtained by comparing full models with reduced models. Treatment position (in the left or right arm of the olfactometer) was included in the odor context-model for old mosquitoes because we observed a slight bias towards one side of the olfactometer (*P* = 0.018). GLMs with a binomial error distribution (logit-link function) were used with the number of mosquitoes attracted to the odor source supplemented with CiJA as the binomial response variable, and the total number of mosquitoes in both capture cages as the binomial total. The concentration of CiJA was included as a factor with three levels. All analyses were performed in R version 3.5.0 (R Core Team 2018).

## Results

### Effect of Cis-jasmone and Methyl Jasmonate on Mosquito Landing

The landing assay showed that *Ae. aegypti* females landed significantly less often on a surface with CiJA and MeJA compared to EtOH (Table 1). Approximately 5.5 landings per mosquito were recorded on the negative control with attractive host-associated cues (odor, heat, and moisture) and a strip incubated in the solvent, ethanol, in both experimental series. Treatment significantly affected the number of landings in both experimental series. At a low concentration (0.1%), only CiJA significantly reduced the number of landings compared to the EtOH control (pairwise comparisons, *P* = 0.013). At a tenfold higher concentration, significantly fewer landings were observed on both CiJA and MeJA compared to the EtOH control. On average less than one landing per mosquito was observed on 1% CiJA (pairwise comparisons, *P* < 0.001), while approximately 2 landings per mosquito were recorded on 1% MeJA (*P* < 0.001).

**Table 1:** Number of landings of *Ae. aegypti* females on surfaces with *cis*-Jasmone (CiJA) and methyl jasmonate (MeJA) compared to ethanol (EtOH) as a negative control

		Number of landings per mosquito (mean ± SE) ^*a*^		
	*P*_treatment_^*b*^	Control (EtOH)	CiJA	MeJA
Low concentration (0.1%)	*P* = 0.034 (*χ*^2^_2_ = 6.75) ^*c*^	5.34 ± 0.41 ^A^	3.27 ± 0.70 ^B^	4.02 ± 0.38 ^AB^
High concentration (1%)	*P* < 0.001 (*χ*^2^_2_ = 26.21) ^*d*^	5.57 ± 0.66 ^A^	0.84 ± 0.42 ^B^	1.97 ± 0.32 ^C^

^*a*^ Landings were observed for 10 min for groups of 4–10 mosquitoes (number of replicate tests: *N* = 8, *N* = 7 at low and high concentration respectively)

^*b*^ Numbers of landings per group of mosquitoes were analyzed per concentration with GLMs assuming a negative binomial distribution. Means followed by different lower-case letters within rows indicate significant pairwise differences between treatments (*P* < 0.05)

^*c*^ Temperature inside the cage significantly influenced the number of landings at low concentration (GLM, *P* < 0.001, *χ*^2^_1_ = 12.56)

^*d*^ The number of mosquitoes per run significantly influenced the number of landings at high concentration (GLM, *P* = 0.018, *χ*^2^_1_ = 5.57)

### Effect of Odor Context and Mosquito Age on Olfactory Response to Cis-Jasmone

**Fig. 1:**
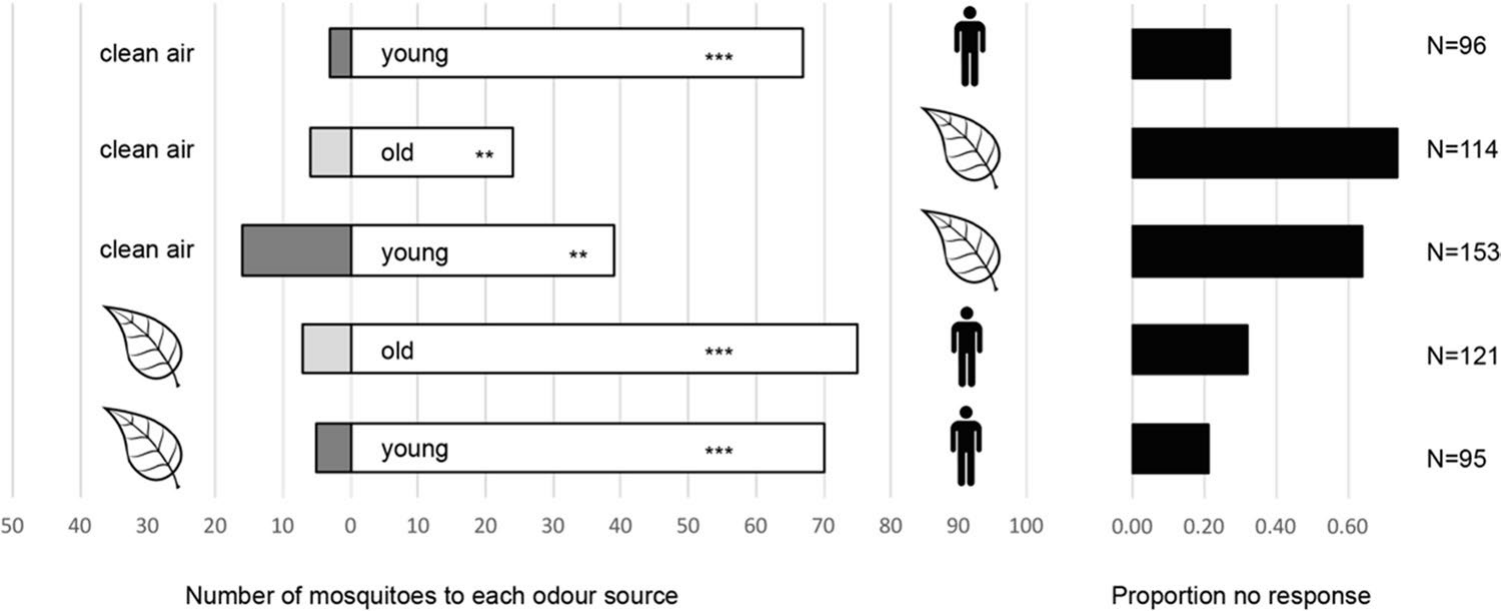
Olfactory response of *Ae. aegypti* females to volatiles from humans or plants in a Y-tube olfactometer. Human volatiles were presented as a worn nylon sock. Plant volatiles were presented as Lima bean leaves. ‘Young’ and ‘old’ inside bars reflect the age of mosquito females, respectively 1–3 d or 7–9 d after adult emergence. Mosquito numbers were summed over 6 replicates with ca. 20 mosquitoes each (range 9–22). Black bars represent the proportion of mosquitoes that did not make a choice. *N* indicates the total number of mosquitoes tested per treatment. Per combination of odor sources, a binomial test was used to evaluate if the choice of mosquitoes differed from a 50:50 distribution (** *P* < 0.01; *** *P* < 0.001)

Control experiments showed that young (1–3 d after adult emergence) *Ae. aegypti* females were strongly attracted to human odor (94 ± 5%, *P* < 0.001, binomial test, Fig. 1); old (7–9 d after emergence) females also responded strongly to human odor (89%, data not shown). Females of both age groups were significantly attracted to the odor of Lima bean leaves (respectively 69 ± 7% and 76 ± 9%, *P* ≤ 0.003) in the Y-tube olfactometer. Both groups of mosquitoes had a strong preference for human over plant odor (respectively 92 ± 4% and 93 ± 4%, *P* < 0.001). Notably, the percentage mosquitoes that chose for either odor source was approximately twice as high in the experiments with human odor as compared to those where only the plant odor was presented (Fig. 1).

**Fig. 2:**
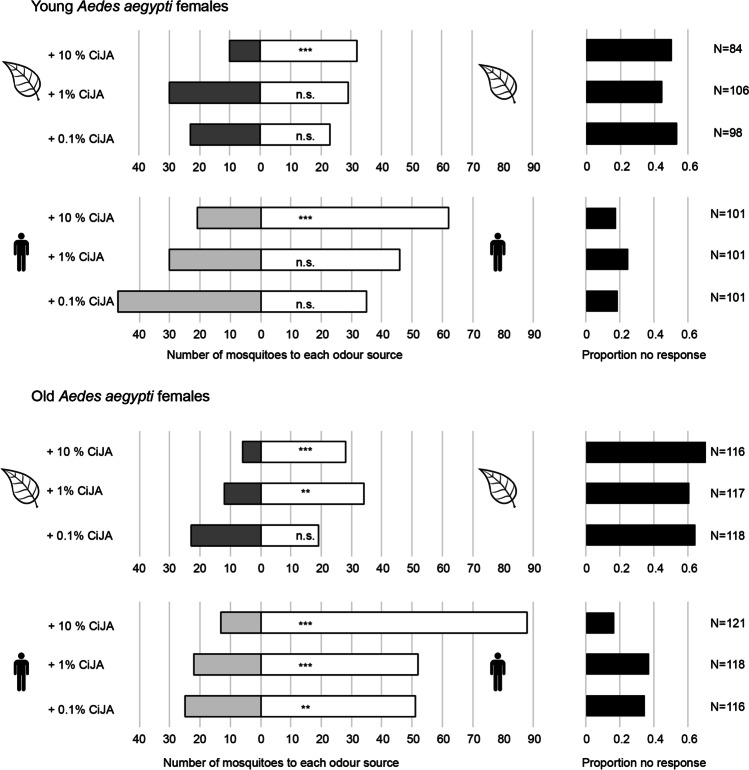
Olfactory response of *Ae. aegypti* females to plant or human odor in combination with *cis*-jasmone (CiJA) in a Y-tube olfactometer. Lima bean leaves or worn socks were presented in both arms of the olfactometer, with CiJA added on one side (at concentrations of 0.1, 1, or 10% in EtOH) and a control (EtOH only) on the other side. Young (1–3 d after emergence) and old (7–9 d after emergence) mosquito females were tested in separate experiments. Grey-shaded bars represent the number of mosquitoes that chose the odor source with CiJA and white bars represent the number of mosquitoes that chose the control odor source. Mosquito numbers were summed over 6 replicates with ca. 20 mosquitoes each (range 13–26). Black bars represent the proportion of mosquitoes that did not make a choice. *N* indicates the total number of mosquitoes tested per treatment. Per combination of odor sources, a binomial test was used to evaluate if the choice of mosquitoes differed from a 50:50 distribution (** *P* < 0.01; *** *P* < 0.001; n.s. *P* > 0.05)

Odor context (plant or human) had no significant influence on the olfactory responses of young *Ae. aegypti* females to CiJA (Fig. 2, GLM, *χ*^2^_1_ = 0.07; *P* = 0.79) and there was no significant interaction between odor context and concentration of CiJA (*χ*^2^_2_ = 2.34; *P* = 0.31). Young *Ae. aegypti* females did not distinguish between the odor sources with or without CiJA (*P* > 0.05, binomial test), except at the highest concentration of CiJA (10%), which led to a preference for the odor sources (plant or human) without CiJA. CiJA concentration indeed significantly affected the olfactory responses of young mosquito females (*χ*^2^_2_ = 24.77, *P* < 0.001).

Old *Ae. aegypti* females appeared more sensitive to CiJA in a context of human odor than in a context of plant odor (Fig. 2). Old females significantly preferred human odor without CiJA at all three concentrations (*P* ≤ 0.004, binomial tests), and plant odor without CiJA at 1% and 10% (*P* ≤ 0.002), while they did not distinguish between plant odor with or without 0.1% CiJA (*P* = 0.64). Nevertheless, the effect of odor context was not significant (GLM, *χ*^2^_1_ = 2.55, *P* = 0.11) and there was no significant interaction between odor context and concentration of CiJA (*χ*^2^_2_ = 4.39, *P* = 0.11). Olfactory responses of old *Ae. aegypti* females were significantly influenced by CiJA concentration (*χ*^2^_2_ = 25.37, *P* < 0.001).

Because odor context did not influence mosquito choice, we next evaluated the effect of mosquito age on the response of *Ae. aegypti* females to CiJA, irrespective of combining CiJA with an attractive human or plant odor. Old females responded significantly more strongly to CiJA than young females (GLM, *χ*^2^_1_ = 16.66, *P* < 0.001). There was no significant interaction between mosquito age and concentration of CiJA (*χ*^2^_2_ = 0.17, *P* = 0.91), while the concentration of CiJA had a strongly significant effect on olfactory responses of *Ae. aegypti* females (*χ*^2^_2_ = 48.07, *P* < 0.001).

## Discussion

We investigated olfactory responses of mosquitoes to jasmonates, plant hormones that have an infochemical function for herbivorous insects and their natural enemies. Methyl jasmonate (MeJA) has a similar structural motif as the well-known synthetic insect repellent DEET. Here, we demonstrate that *Ae. aegypti* females avoid MeJA and CiJA as well. We further show that CiJA is avoided by mosquito females in the context of both attractive plant and human odors. This finding suggests that jasmonates are inherently aversive cues to mosquitoes, independent of the odor context in which they are presented. Our experiments showed that the olfactory response to CiJA was influenced by mosquito age because older (7–9 d) *Ae. aegypti* females avoided CiJA at a lower concentration than younger females (1–3 d).

Both volatile jasmonates (MeJA and CiJA) reduced the number of times *Ae. aegypti* females landed on an attractive surface in our experiments. This was expected because jasmonates were known to repel ticks and mosquitoes (Garboui et al. 2007; Zeng et al. 2018), and because MeJA was postulated to be the natural ligand of the olfactory receptor that is activated by DEET in the southern house mosquito *Cx. quinquefasciatus* (Xu et al. 2014). Interestingly, extending our experiments to a choice situation in a Y-tube olfactometer showed that CiJA appears to be detected by *Ae. aegypti* at much lower doses than those that affected mosquito behavior in the landing assay. In these choice experiments, a 20-fold lower amount was applied on filter paper as compared to the amount impregnated in the nylon material in the landing assay. Mosquito females preferred odor sources without additional CiJA or did not discriminate between the two options, depending on the concentration of CiJA and mosquito age.

Avoidance of sources of volatile jasmonates likely originates from a function in the mosquito – plant relationship. Mosquito feeding on plant fluids is ancient, and blood-feeding may have evolved from phytophagy (Lehane 2005; Peach and Gries 2020). Nectar is an important source of nutrition for extant blood-feeding mosquito taxa, particularly for males and for young females before they take their first bloodmeal (Barredo and DeGennaro 2020; Foster 1995; Foster and Takken 2004). We expected young sugar-deprived females to be attracted to plant odor regardless of the presence of additional CiJA, while older females that had already sugar-fed would be more selective. Our findings are in agreement with these expectations because mosquito age significantly influenced their olfactory response to CiJA, irrespective of the odor context. Older *Ae. aegypti* females avoided CiJA at lower concentrations than young females, perhaps because they are more risk-aversive when they have not yet reproduced (see below). Few studies to date have investigated whether mosquito age influences their responses to aversive or repellent compounds, but our results are in line with a recent report that showed that the effectiveness of DEET increased with mosquito age in females of *An. gambiae* and *Ae. albopictus* (Mulatier et al. 2018). Alternatively, our findings may be explained by an effect of mosquito age on their response to attractive odors, and on how selective these responses are. The olfactory response to host-derived cues is indeed regulated by mosquito age in *Ae. aegypti* and this process is mediated by changes in the expression of chemosensory-related genes in the antennae (Tallon et al. 2019).

Elevated levels of jasmonates are associated with plants under herbivore attack because these compounds play a key role in plant defense against herbivores (Gfeller et al. 2010; Wasternack 2015). The jasmonic acid pathway mediates mechanisms such as increased emission of volatile organic compounds (Pickett et al. 2007; Turlings and Erb 2018) and increased production of (extrafloral) nectar, for example in Lima bean plants (e.g. Kost and Heil 2006). This activation of herbivore-induced direct and indirect defense mechanisms is associated with reduced attractiveness to herbivores and increased attractiveness to natural enemies of herbivores (e.g. Bruce et al. 2008). We found that *Ae. aegypti* females avoid the odor of Lima bean plants with additional CiJA or did not distinguish between the two odor sources. This finding suggests that an elevated level of CiJA signals a less profitable environment to *Ae. aegypti* females. A plausible explanation for this result is that plants with elevated levels of jasmonates are associated with the increased attraction of herbivore natural enemies that may also prey on mosquitoes, thereby acting as a signal of ‘enemy-dense space’. Documented mosquito predators on plants are crab spiders (Peach and Gries 2016) and a salticid spider that uses terpenoid plant odors in its foraging behavior (Carvell et al. 2017).

To summarize, our Y-tube olfactometer experiments demonstrate that avoidance of the volatile plant hormone CiJA by *Ae. aegypti* is mediated by olfaction. Our findings strengthen the idea that avoidance of jasmonates by mosquitoes results from their interactions with plants, possibly because jasmonates signal a higher risk of predation. Experiments on predation risks of mosquitoes during nectar-feeding are clearly necessary to address this topic further. Ultimately, a better understanding of mosquito responses to jasmonates may contribute to understanding the evolutionary significance of avoidance of herbivore-induced volatile plant compounds by mosquitoes in the context of mosquito—plant interactions.

## Data Availability

Data will be archived in a digital repository upon acceptance.
